# Cinobufagin-Loaded and Folic Acid-Modified Polydopamine Nanomedicine Combined With Photothermal Therapy for the Treatment of Lung Cancer

**DOI:** 10.3389/fchem.2021.637754

**Published:** 2021-03-29

**Authors:** Jianwen Li, Zhanxia Zhang, Haibin Deng, Zhan Zheng

**Affiliations:** ^1^ Department of Oncology, Longhua Hospital, Shanghai University of Traditional Chinese Medicine, Shanghai, China; ^2^ Cancer Institute, Longhua Hospital, Shanghai University of Traditional Chinese Medicine, Shanghai, China

**Keywords:** anticancer nanomedicine, photothermal therapy, targeted delivery, stimuli response, biodegradation

## Abstract

Cinobufagin is used as a traditional Chinese medicine for cancer therapy. However, it has some disadvantages, such as poor water solubility, short circulating half-life, and low bioavailability. In the present study, a targeted delivery and smart responsive polydopamine (PDA)-based nanomedicine for delivering cinobufagin was rationally designed to improve the anticancer efficacy of the compound for the treatment of lung cancer. The modification of the nanomedicine using folic acid first mediated tumor targeting via the interaction between folic acid and its receptors on tumor cells. After lysosomes escape, the PDA nanomedicine was triggered by the low pH and released its cargo into the tumor microenvironment. The nanomedicine had a better therapeutic effect against lung cancer when used in combination with photothermal therapy. Compared with other nanomedicines used with photothermal therapy, this nanocarrier was not only sensitive to biologically low pH levels for on-demand drug release, but was also biodegradable, breaking down into biocompatible terminal products. Therefore, the proposed drug delivery system with targeted delivery and smart release demonstrated potential as a multifunctional nanoplatform that can enhance the bioavailability and reduce the side effects of chemotherapeutic agents.

## Introduction

Malignant tumors pose a major threat to human health and are characterized by rapid growth, strong metastasis, and high recurrence rate (Quail and Joyce, 2013). In addition, their morbidity and mortality rates are on the rise (Siegel et al., 2020). Although many chemotherapeutic agents used clinically can inhibit tumor growth significantly, they have various toxic and side effects, and have a low drug utilization rate (Kroschinsky et al., 2017). Therefore, new therapies need to be developed to circumvent these issues.

Photothermal therapy (PTT) is a novel noninvasive tumor treatment strategy that can transform near-infrared (NIR) light into heat using organic photosensitive molecules or inorganic nanomaterials (Hussein et al., 2018). The main advantages of PTT for treating cancer include thermal ablation, reversal of drug resistance, and inhibition of tumor metastasis (Jiang et al., 2015). Dong et al. developed a new method to efficiently produce Mo-based POM using β-Mo_2_C as the raw material, and revealed its REDOX cycle behavior in the tumor microenvironment, and successfully applied it to second near-infrared window (NIR-II) photoacoustic imaging-mediated photothermal and chemodynamic synergistic therapy (Liu et al., 2019). At present, organic photosensitive molecules mainly include indocyanine green (ICG) and methylene blue (Fan et al., 2020). However, the blood circulation half-life of these compounds is short, and they cannot be enriched selectively in the tumor area (Gonçalves et al., 2020). Although a large variety of nanomaterials, including noble metal nanoparticles (NPs), chalcogenide nanomaterials, carbon nanomaterials, and quantum dots, have been reported to exhibit photothermal activities, their poor biocompatibility limits their application in PTT (Zhang et al., 2016; Wang et al., 2020). Recently, polydopamine (PDA), derived from the self-polymerization of dopamine, has received attention as a biocompatible (natural melanin) photothermal material as well as a nanocarrier containing amino groups to facilitate surface modification (Farokhi et al., 2019).

Nanomedicine has the advantages of enhanced permeability and retention (EPR), extending the biological half-life of drugs, targeted delivery, and controlled release, even across the blood brain barrier (Zhao et al., 2020). To date, the FDA has approved various nanomedicines, such as doxil (liposome doxil), abraxane (albumin paclitaxel), and onivyde (liposome irityde) for treating cancer (Gaitanis and Staal, 2010). Although nanomedicines have achieved some success in anti-tumor therapy, the safety and toxicity of nanocarriers need to be evaluated and their clinical efficacy needs to be improved. Recent studies have found that the modification of nanomedicines by targeting molecules on their surface (positive targeting) can improve the specific delivery of these nanomedicines at tumor sites (Hill and Mohs, 2016). Targeting molecules usually include small molecules such as folic acid (FA), lectin, peptides, polysaccharides, antibodies, and nucleic acid aptamers (Rashidi et al., 2016). Previous studies demonstrate the use of RGD polypeptide, cetuximab, and EGFR (epidermal growth factor receptor) aptamer, all of which have targeted effects on tumor tissues (Wang et al., 2016; Narmani et al., 2019; Zhang et al., 2019). Overexpression of the FA receptor (FR) in various tumor cells makes it a good candidate for targeted delivery of nanomedicine (Zhang et al., 2020). The new generation of nanomedicine not only has the advantage of targeted delivery but can also release the cargo, according to the stimulation of the tumor microenvironment. Many smart nanomedicines that respond to external stimuli, such as light, magnetic field, and ultrasound, as well as internal stimuli, such as pH, temperature, enzyme, and redox potential responses have been explored (Zhou et al., 2018). Previous studies have demonstrated the release of nanomedicines in response to redox potential, DNAzyme, nuclease, crown ether, visible–ultraviolet light, and autophagy-lysosome processes (Zhang et al., 2013a; Zhang et al., 2013b; Zhang et al., 2013c; Zhang et al., 2014; Wang et al., 2016; Narmani et al., 2019). A large number of studies have shown that the pH level of tumor tissues (6.5–6.9) is generally lower than that of para-carcinoma tissue (7.2–7.4), which is mainly due to the anaerobic metabolism (Warburg effect) of tumor tissues and the production of a large number of acidic metabolites, such as lactic acid (Liu et al., 2014). Although there have been some reports on pH-responsive nanomedicines (Tang et al., 2018), there are only a few studies on biodegradable nanomedicines, such as PLGA (poly(lactic-co-glycolic acid), cyclodextrin, and chitosan, as well as treatment in combination with PTT (Zhang et al., 2018), which markedly restricts the prospects of nanomedicines.

The main components of cinobufagin (Cino) are indole alkaloids, which are extracted from the dry epidermis of *Bufo gargarizans* Cantor or *B. melanostictus* Schneider (Xie et al., 2012; Dai et al., 2018). Cino aids in clearing away heat and detoxification, relieving pain, relieving swelling, and removing stasis. According to the “Compendium of Materia Medica” the smell of Cino is symplectic cool, and it has the effect of clearing fever and damp elimination. Currently, Cino is used as a traditional anti-tumor medicine in China. It has shown significant efficacy in the treatment of various malignant tumors, especially lung, liver, and pancreatic cancers, when used alone or in combination with other chemotherapy drugs (Qi et al., 2011). However, due to the poor water solubility, short circulating half-life, and low bioavailability of Cino, improving its anticancer efficacy is an urgent clinical problem that needs to be solved (Ren et al., 2019).

**FIGURE 1:**
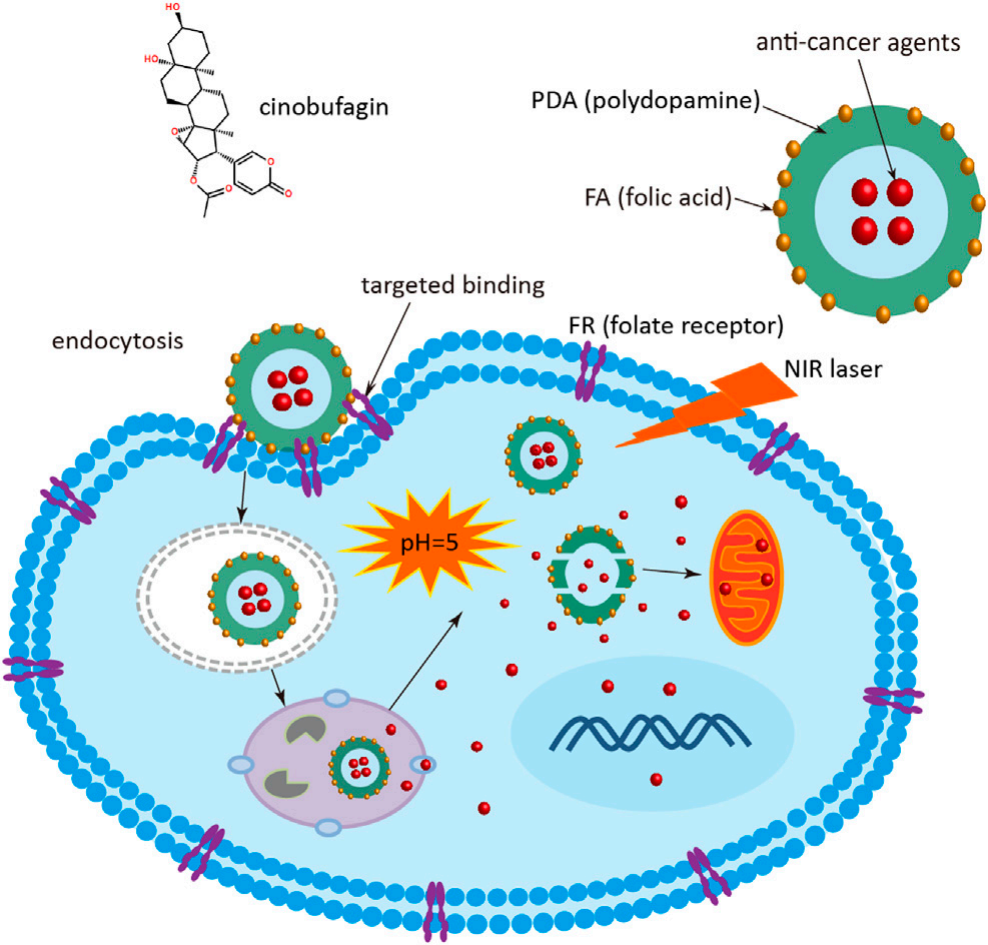
Schematic diagram of Cino nanomedicine with targeted delivery and smart response. Cino, cinobufagin; PDA, polydopamine; FA, folic acid; FR, folate receptor; NIR, near infrared.

In order to improve the therapeutic efficacy and enhanced solubility of the anticancer agent, a stimuli-responsive and targeting molecule-modified organic nanomedicine was developed. The biodegradable PDA nanomedicine was firstly synthesized via a classical Stöber method (by the reduction of dopamine hydrochloride to PDA in an aqueous alkaline solution) (Ren et al., 2019). Subsequently, the surface of the PDA nanomedicine was modified by the targeting molecule FA through the covalent coupling reaction of amino and carboxyl groups in the presence of EDC and NHS. The process of delivering the nanomedicine is described in Figure 1. At the beginning, the FA-modified nanomedicine is recognized by FR, which is highly expressed in tumor cells. The nanomedicine is then delivered into the cytoplasm via the endosome through FA and FR-mediated endocytosis. After lysosome escape, the nanomedicine is delivered into the cell cytoplasm. Then the PDA nanomedicine is stimulated by the low intracellular pH due to the accumulation of acidic metabolites (e.g., lactic acid) produced by the high-rate of anaerobic glycolysis in tumor cells, and the anticancer agent Cino is released for inhibiting the proliferation of cancer cells. Finally, the PDA nanomedicine possesses a better therapeutic effect when combined with PTT.

## Materials and Methods

### Materials

Cino was purchased from Absin. Dopamine hydrochloride, doxorubicin (DOX), and FA were purchased from Aladdin. N-(3-Dimethylaminopropyl)-N′-ethylcarbodiimide hydrochloride (EDC) and N-hydroxysuccinimide (NHS) were purchased from Sigma. All other organic solvents used in this study were of an analytical grade. Cleaved caspase-3 antibody was purchased from Abcam. Cell counting kit-8 (CCK-8) was purchased from MedChemExpress. Alanine transaminase (ALT), aspartate aminotransferase (AST), and creatinine (CRE) activity assay kits were purchased from the Nanjing Jiancheng Bioengineering Institute.

### Synthesis of Anticancer Agent-Loaded Polydopamine Nanomedicine

A classical Stöber method with some modifications was used to synthesize the PDA nanomedicine (Bao et al., 2018). Briefly, a mixture of 290 ml of ultrapure water, 110 ml of ethanol, and 1.5 ml of NH_4_OH was stirred at room temperature for 30 min. Then, 50 mg of Cino or 50 mg of DOX in 10 ml of ethanol was added to the above mixture. Subsequently, 0.5 g of dopamine hydrochloride in 10 ml of ultrapure water was added, and the reaction was stirred overnight. Finally, the nanomedicine was collected by centrifugation to remove the unloaded drug at 10,000 rpm for 10 min, washed twice with ultrapure water using a centrifuge at room temperature, and dried overnight on a lyophilizer.

As for the modification using FA, the obtained nanomedicine (100 mg) was first dispersed in 10 ml of phosphate buffered saline (PBS) (10 mM, pH 6.0). Subsequently, 50 mg of FA in 1 ml of ultrapure water was added to the above mixture. Next, 50 mg of EDC and 50 mg of NHS were added, and the mixture was stirred for 2 h. Finally, the FA-modified nanomedicine was collected by centrifugation at 5,000 rpm for 10 min, washed twice with ultrapure water using a centrifuge at room temperature, and dried overnight. Similarly, the blank PDA NPs were synthesized using the above-mentioned method, except for the addition of the anticancer agent.

### Characterization of Polydopamine Nanoparticles

Transmission electron microscopy (TEM) and dynamic light scattering (DLS) were used to characterize the PDA NPs. Aliquots of PDA NP suspension were first dispensed onto parafilm sheets in a humidified Petri dish, and the vesicles were deposited on a carbon-coated grid (300-mesh) for 3 min. Subsequently, the grids were analyzed using a TEM (JEM-1230, JEOL). For DLS studies, the size distribution and zeta potential of the NPs were analyzed using a Malvern ZetaSizer Nano ZS90 particle size analyzer.

### Assessment of Encapsulation Efficiency and Loading Content of Cino in the Polydopamine Nanomedicine

To measure the Cino content in the Cino-loaded PDA nanomedicine, the nanomedicine was diluted in acetonitrile. Subsequently, the concentration of Cino in the samples was determined using a Waters Acquity UPLC apparatus equipped with a Waters Acquity UPLC HSS T3 (2.1 × 100 mm, 1.8 μm) chromatographic column. The mobile phase consisted of acetonitrile (A) and 0.1% formic acid (B). The gradient elution program was as follows: 0–19 min, 10–95% (A); 19–20 min, 95–100% (A); 20–21 min, 100–10% (A); and 21–25 min, 10% (A). The other parameters were flow velocity: 0.4 ml/min; column temperature: 40°C; and sample volume: 2 μL. The monitoring wavelength range was 190–800 nm. The amount of Cino in the PDA nanomedicine was measured at 254 nm using a standard curve (absorbance vs. concentration). Cino encapsulation efficiency was 21.3% and was calculated as the ratio of the amount of Cino encapsulated in the NPs to the total amount of Cino fed for encapsulation. Cino loading content in the PDA nanomedicine was 9.1% and was calculated as the ratio of the amount of Cino encapsulated in the NPs to the total amount of NPs including Cino. Loading content of DOX in the PDA nanomedicine (dissolved in Tris buffer (pH = 9.0) for 20 min) for targeted delivery and pH release was measured using a nano-drop UV-Vis spectrophotometer at 539 nm using a standard curve (absorbance vs. concentration). The excitation and emission spectra of DOX is shown in Suplementary Figure S5 (Supplementary Material).

### Cell Culture and Cell Viability

Human lung/brunch normal epithelial (Beas2B), human lung adenocarcinoma (A549), and Lewis lung carcinoma (LLC) cell lines were purchased from the Cell Center of the Chinese Academy of Medical Sciences. Beas2B and LLC cells were cultured in DMEM (Hyclone, Logan, UT), and A549 cells were cultured in RPMI 1640 medium (Hyclone, Logan, UT), containing 10% fetal bovine serum (Biochrom AG, Berlin, Germany) and 1% penicillin-streptomycin solution at 37°C with 5% carbon dioxide.

For the cell viability assay, the cells were plated in 96-well plates at a density of 2 × 10^3^ cells per well in quadruplicate and cultured overnight. After incubation with different reagents for 48 h, the cells were subjected to the CCK-8 assay according to the manufacturer’s specifications.

### Targeted Effect of Folic Acid -Modified Nanomedicine

For analysis of the targeted effect of FA, the Beas2B, A549, and LLC cells were first seeded in confocal dishes at 2 × 10^5^ cells per well and cultured for 24 h. The cells were then incubated with 0.5 mg/ml of DOX-loaded FA-modified PDA nanomedicine for 4 h. Subsequently, the samples were washed with PBS and fixed with 4% paraformaldehyde for 30 min. Next, 0.5 μg/ml of Hoechst 33,258 was used to stain the cell nuclei for 5 min after washing with PBS. Finally, the targeted effect of FA was observed using a fluorescence microscope (Leica) at the red channel, after washing with ultrapure water and drying.

For analysis of the targeted effect of FA using a flow cytometry, the Beas2B, A549, and LLC cells were first seeded in 6-well plates at 2 × 10^5^ cells per well, and cultured for 24 h. The cells were then incubated with 0.5 mg/ml of DOX-loaded FA-modified PDA nanomedicine for 4 h. Finally, the cells were harvested and analyzed on a flow cytometer (BD) at the PE (red) channel.

### Stimuli Response of Doxorubicin-Loaded Polydopamine Nanomedicine

For the pH-responsive release of the PDA nanomedicine, Cino-loaded nanomedicine was used to stimulate the release of the PDA nanomedicine. First, 2.5 mg of Cino-loaded PDA nanomedicine was dissolved in 5 ml of 1 × PBS buffer (pH = 7.4) and divided into five portions of 1 ml each for use as control (pH = 7.4). Simultaneously, 5 mg of Cino-loaded PDA nanomedicine was dissolved in 10 ml of 1 × PBS buffer (pH = 5.0) and divided into ten portions of 1 ml each. Five of them were used as the pH (pH = 5.0) release group, and the other five were used as the pH (pH = 5.0) release with laser irradiation (808 nm, 2 W cm^−2^, 5 min) group. Then, three samples from the different groups were precipitated at predetermined time intervals, and the content of Cino in the supernatant was determined using the UPLC apparatus.

### *In Vitro* Anti-Tumor Efficacy of Cino-Loaded Polydopamine Nanomedicine in Lung Cancer Cells

For the cell viability assay, Beas2B, A549, and LLC cells were seeded in 96-well plates at 2 × 10^3^ cells per well in quadruplicate and cultured overnight. After incubation with different concentrations of free Cino, Cino-loaded PDA nanomedicine, and Cino-loaded PDA nanomedicine with laser irradiation (808 nm, 2 W cm^−2^, 5 min) for 48 h, the cells were subjected to CCK-8 assay according to the manufacturer’s specifications.

### *In Vivo* Anti-Tumor Efficacy of Cino-Loaded Polydopamine Nanomedicine

To establish xenograft tumors, six-week-old male nude mice (weighing approximately 20 g) were purchased from the Sippr-BK Laboratory Animal Co. Ltd (Shanghai, China). They were randomly divided into five groups (*n* = 6 for each group) and subcutaneously injected with 2 × 10^5^ of LLC cells on the left side of the armpit. The length and width of tumors were measured using calipers every two days, and the tumor volume was calculated as (length × width^2^)/2.

To evaluate the anticancer activity of the Cino-loaded PDA nanomedicine *in vivo*, the mice were intraperitoneally injected with saline, blank PDA NPs (∼5 mg/kg), free Cino (1 mg/kg), Cino-loaded nanomedicine (at a Cino dose of 1 mg/kg), and Cino-loaded nanomedicine (at a Cino dose of 2 mg/kg) with laser irradiation (808 nm, 2 W cm^−2^, 5 min), and treated every two days after the tumor volume reached approximately 50 mm^3^. All mice were sacrificed, and their tumor weights and gross volumes were measured when the largest tumor volume was less than 800 mm^3^. In addition, orbital blood obtained before mice sacrifice was mainly used for the detection of hepatorenal function. The tumor tissues were fixed in formalin for immunohistochemical analyses.

### Immunohistochemical Staining

Tumors in each group were fixed with 5 ml of formalin overnight, dehydrated in ethanol, embedded in paraffin, and sectioned (at a thickness of 5 μm). Next, slides were deparaffinized in xylene and ethanol, and rehydrated in water. Subsequently, antigen retrieval was performed by heating in a microwave for 30 min in sodium citrate buffer (pH = 6.0). Slides were then quenched in hydrogen peroxide (3%) to block endogenous peroxidase activity and washed with TBST buffer. Finally, the primary antibodies were incubated at 4°C overnight, followed by the use of a SuperPicture™ Polymer Detection kit (Life Technologies) according to the manufacturer’s instructions, along with antibodies against cleaved caspase-3 (Abcam).

### Statistical Analysis

Data are presented as mean ± standard deviation (SD). Statistically significant differences between two groups were analyzed by hypothesis testing with the two-sample *t*-test, and indicated by *p < 0.05, **p < 0.01, and ***p < 0.001; *p* < 0.05 was considered statistically significant in all analyses (95% confidence level).

## Results and Discussion

### Synthesis and Characterization of Polydopamine Nanoparticles

**FIGURE 2:**
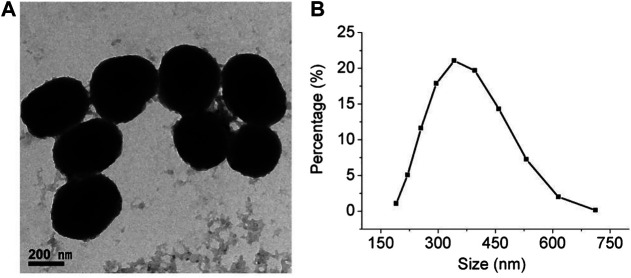
Characterization of synthesized PDA NPs. **(A)** TEM image of PDA NPs (scale bar = 200 nm). **(B)** Particle size distribution using dynamic light scattering (DLS).

A classical Stöber approach was used to prepare the PDA NPs. During the reaction, the mixture quickly turned from colorless to black, indicating the formation of PDA NPs, because dopamine is a natural melanin. TEM and DLS were employed to characterize the morphology and size of the PDA NPs. As shown in Figure 2A, the TEM image indicates that the PDA NPs possessed a spherical and uniform morphology. As shown in Figure 2B, the DLS image correlates well with the TEM results, and the size of most of the NPs was approximately 330 nm.

The zeta potentials of the blank PDA NPs and Cino-loaded nanomedicine were measured using DLS. As shown in Supplementary Figure S1 (Supplementary Material), the zeta potentials of the PDA NPs and Cino-loaded nanomedicine were −42 mV and −31 mV, respectively, indicating that Cino was successfully embedded in the PDA nanomedicine. The negative charge may be caused by the modification of FA on the surface of the nanomedicine.

Furthermore, Fourier transform infrared spectroscopy (FITR) of PDA NPs, FA, and FA-modified PDA NPs were recorded. As shown in Supplementary Figure S2 (Supplementary Material), the characteristic peak of FA (at 1700 cm^−1^) can be observed in FA-modified PDA NPs, revealing that FA was successfully modified on the surface of the PDA NPs.

The absorption spectrum and the photostability of the PDA NPs were also measured. As shown in Supplementary Figure S3 (Supplementary Material), the PDA NPs possess broad absorption in the NIR region at 750–900 nm, exhibiting the capability to convert NIR into heat similar to other photothermal materials (Ding et al., 2019). Importantly, the PDA NPs have excellent photothermal stability and repeatability in which the conversion efficiency was virtually unchanged after five rounds of ON/OFF irradiation cycles. These results indicated that the PDA NPs had good photothermal conversion efficiency and photothermal stability. Thus, the NPs were suitable for use as PTT agents.

### Biocompatibility of the Blank Polydopamine Nanoparticles

PDA, a natural melanin, was chosen as the basic component to guarantee the favorable biocompatibility of the nanocarrier. In theory, the blank PDA NPs should have excellent biocompatibility because of the biodegradable metabolites homovanillic acid and trihydroxyphenylacetic acid (Marín-Valencia et al., 2008). As shown in Supplementary Figure S4 (Supplementary Material), blank PDA NPs showed no obvious cytotoxicity to Beas2B, A549, or LLC cells, indicating that the PDA NPs are biocompatible.

In this study, PDA is not only used as the nanomedicine carrier but also has the property of PTT. Hence, we also explored the influence of the 808 laser on cell growth. As shown in the last panel of Supplementary Figure S4 (Supplementary Material), the 808 laser used at 2 W cm^−2^ for 5 min had little effect on the proliferation of Beas2B, A549, and LLC cells. Furthermore, the *in vitro* anti-tumor efficacy of the blank PDA NPs with NIR treatment in lung cancer was also recorded. As shown in Supplementary Figure S6 (Supplementary Material), the cell viability of A549 cells and LLC cells had some influence after incubation with the blank PDA NPs with NIR treatment, indicating that the NPs had the property of photothermal therapy.

### Targeted Delivery of Folic Acid

**FIGURE 3:**
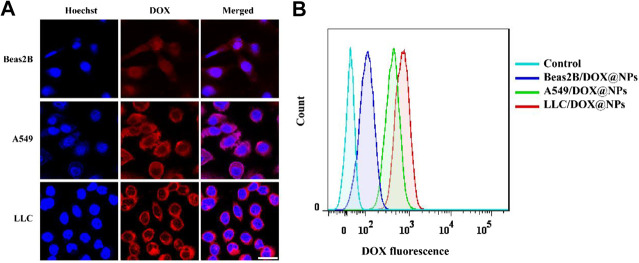
Targeted effect of DOX-loaded FA-modified nanomedicine. **(A)** Fluorescence microscopy images of normal Beas2B, lung cancer A549, and LLC cells after treatment with DOX-loaded FA-modified nanomedicine (0.5 mg/ml) for 4 h. The red color indicates DOX, and the blue color indicates Hoechst; scale bar = 25 μm. **(B)** Flow cytometry analysis of the above samples at PE (red) channel.

In order to improve the efficacy and reduce the side effects of the nanomedicine, FA was used as the targeting molecule for modifying the surface of the PDA nanomedicine. For verifying the targeted effect of FA, DOX-loaded FA-modified PDA nanomedicine was incubated with Beas2B, A549, and LLC cells due to the optical signal of DOX (Chen et al., 2018). As depicted in Figure 3A, fluorescence microscopy indicated the presence of a small amount of PDA nanomedicine around the Beas2B cells. The red color around the Beas2B cells might be due to nonspecific adsorption of the PDA nanomedicine, like the right (red) shift of Beas2B cells observed in the flow cytometry analysis (Figure 3B). In contrast, there was a large amount of targeted adsorption of the nanomedicine around the A549 and LLC cells, suggesting the excellent targeted effect of FA in tumor cells. As depicted in Figure 3B, flow cytometry analysis is consistent with the results of fluorescence microscopy. These data demonstrated that the modification by FA as a targeting molecule can help deliver anticancer drugs specifically to tumor cells.

### Controlled Release and Optothermal Response of the Polydopamine Nanomedicine

**FIGURE 4:**
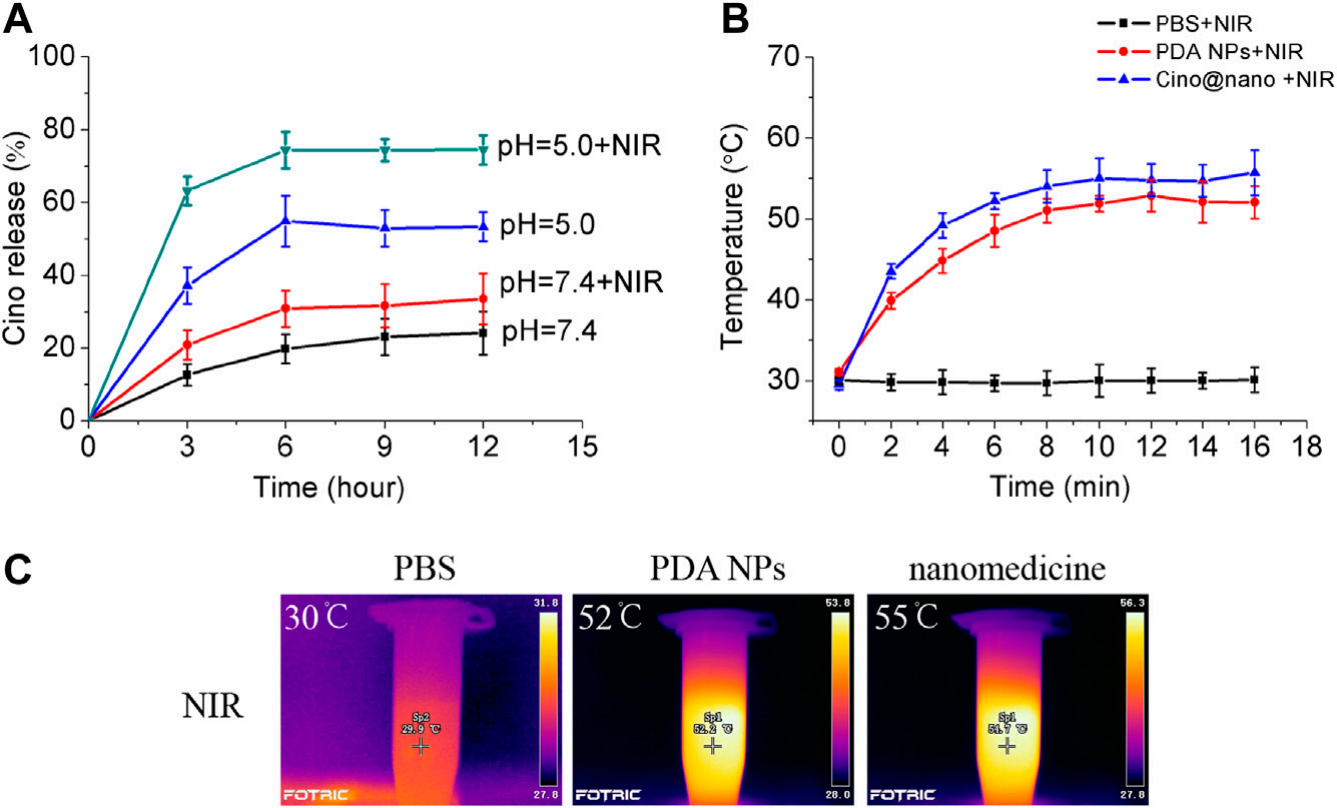
Controlled release and optothermal response of the PDA nanomedicine. **(A)** Cino release from Cino-loaded PDA nanomedicine (0.5 mg/ml) after treatment with pH = 5.0 PBS buffer, without or with an 808 laser (2 W cm^−2^, 5 min). Data are presented as the mean ± SD (standard deviation, *n* = 3). The temperature curves **(B)** and pictures **(C)** of PBS buffer, blank PDA NPs, and Cino-loaded PDA nanomedicine after treatment with the 808 laser.

A large number of studies have shown that the pH of tumor tissues (6.5–6.9) is generally lower than that of normal tissues, which is mainly due to the aerobic glycolysis (Warburg effect) of tumor tissues, and the production of a large number of acidic metabolites, such as lactic acid (Marín-Valencia et al., 2008). The pH-responsive release and optothermal response of the Cino-loaded PDA nanomedicine were studied under low pH conditions (pH = 5.0) with NIR laser irradiation. As shown in Figure 4A, the release curve showed that only 24% of Cino was released in the control group (pH = 7.4 of PBS buffer). The minor leakage of Cino was caused by the relative stability of the PDA nanocarrier under normal conditions. The Cino release reached up to 33% with NIR laser treatment. Importantly, the Cino release reached up to 53 and 74% without and with NIR laser treatment, respectively, at pH = 5.0 within 12 h. The pH-dependent release may be due to the pH sensitivity of the PDA nanocarrier (Crayton and Tsourkas, 2011). After treatment with the 808 laser (2 W cm^−2^, 5 min) at pH = 5.0, the temperature of the PDA nanomedicine was gradually increased, which led to a significant increase in cumulative Cino release. In addition, most of the anticancer agents were released from PDA nanomedicine during the first 6 h. These results confirmed that the PDA nanomedicine could respond to low pH levels and be triggered for on-demand drug release. Further, the laser treatment (808 nm) can accelerate the release of the anticancer agents from PDA nanomedicine.

Next, we explored the photothermal response of the PDA nanomedicine. thermographic images and curves of the PBS buffer, blank PDA NPs, and Cino-loaded PDA nanomedicine after treatment with the 808 laser are shown in Figure 4B. The temperature of the PBS buffer did not change when treated with the 808 laser. However, the temperature of the blank PDA NPs and Cino-loaded PDA nanomedicine increased over time and reached a saturation state; the rate of increase in temperature was proportional to the power of the laser. Furthermore, the temperature curves of the blank PDA NPs and Cino-loaded PDA nanomedicine showed similar trends. Collectively, the efficacy of Cino-loaded PDA nanomedicine was found to be enhanced by PTT in lung cancer therapy.

### *In Vitro* Inhibitory Effect of Polydopamine Nanomedicine on Lung Cancer Cells

**FIGURE 5:**
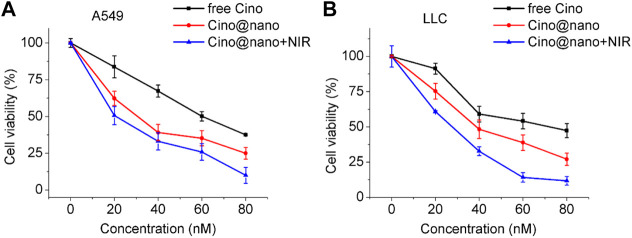
*In vitro* anti-tumor efficacy of Cino-loaded PDA nanomedicine in lung cancer cells. **(A)** Viability of A549 cells after incubation with various concentrations of free Cino, Cino-loaded PDA nanomedicine, and Cino-loaded PDA nanomedicine with NIR treatment. Data are presented as mean ± SD (standard deviation, *n* = 4). **(B)** Viability of LLC cells after incubation with various concentrations of free Cino, Cino-loaded PDA nanomedicine, and PDA nanomedicine with NIR treatment (2 W cm^−2^, 5 min). Data are presented as the mean ± SD (standard deviation, *n* = 4).

To explore the anticancer effect of the PDA nanomedicine, lung cancer cells were incubated with free Cino (control group), Cino-loaded PDA nanomedicine, and Cino-loaded PDA nanomedicine with laser irradiation at 808 nm. As shown in Figure 5A, the half maximal inhibitory concentration (IC_50_) of free Cino was 61 nM in A549 cells. Conversely, the IC_50_ of Cino-loaded nanomedicine was only 32 nM, which is almost half. The IC_50_ of Cino-loaded nanomedicine combined with laser irradiation at 808 nm was even lower, at 21 nM. These results revealed that PDA nanomedicine has a better inhibition potential than its free drug form. In addition, PDA nanomedicine combined with laser irradiation at 808 nm possesses the potential for PTT. Concurrently, the inhibitory effects of the PDA nanomedicine on lung cancer LLC cells were also assessed. As shown in Figure 5B, the IC_50_ of free Cino was 74 nM, whereas that of the PDA nanomedicine was 39 nM. The IC_50_ of the nanomedicine combined with laser irradiation at 808 nm was 31 nM, indicating that the PDA nanomedicine, used along with laser irradiation at 808 nm, has the ability to greatly suppress the proliferation of LLC cells, not only with its chemotherapeutic agents, but also with PTT. The PDA nanomedicine has a better inhibitory effect, which might be due to the different methods of cellular uptake for these agents. For example, free Cino enters cells via diffusion, whereas the PDA nanomedicine enters through endocytosis. In this aspect, endocytosis seems more efficient for carrying a high amount of Cino in contrast to simple diffusion through the cell membrane (Rashidi et al., 2016). Importantly, the PDA nanomedicine posed a much higher selective cytotoxicity to the lung cancer cells, having high FR expression and lower pH than normal cells. In addition, treatment with laser irradiation at 808 nm showed an inhibitory effect on lung cancer cells mainly due to thermal ablation (Li et al., 2018). All the observations demonstrated that the PDA nanomedicine with targeted delivery and controlled release had the best therapeutic effect in the presence of laser irradiation at 808 nm.

### *In Vivo* Anti-Tumor Activities of the Polydopamine Nanomedicine

We firstly investigated the *in vivo* targeted delivery capacity of the PDA nanomedicine. *In vivo* biodistribution of free ICG, ICG-loaded PDA nanomedicine, and ICG-loaded PDA nanomedicine with FA modification was monitored using an *in vivo* imaging system due to the NIR fluorescence signal of ICG (Li et al., 2018). As shown in Supplementary Figure S7 (Supplementary Material), only weak ICG fluorescence in the tumor could be visualized for the free ICG group at 24 h post-injection. In contrast, the fluorescence intensity of PDA nanomedicine without FA modification in the tumor tissues was still very strong. However, strong ICG fluorescence in the liver was observed, indicating that PDA nanomedicine without FA modification could prolong systemic circulation in blood due to the EPR effect. Importantly, the ICG fluorescence of PDA nanomedicine with FA modification was much stronger at the tumor site than that of PDA nanomedicine without FA modification, and weaker in the liver, demonstrating a good tumor-targeting ability. These results reveal that PDA nanomedicine with targeted modification could improve the therapeutic effect and reduce toxic and side effects.

**FIGURE 6:**
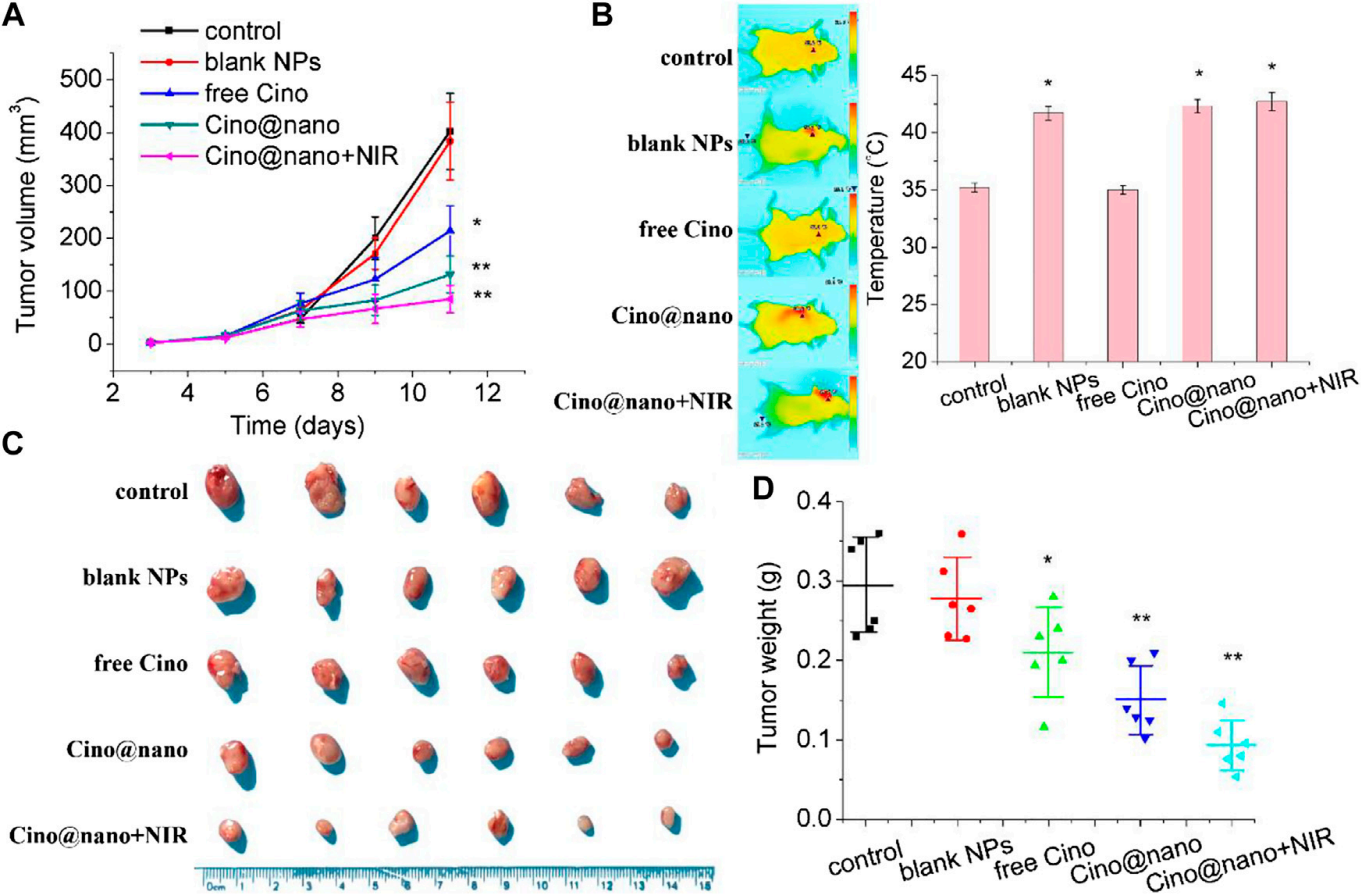
*In vivo* anti-tumor efficacy of Cino-loaded PDA nanomedicine. **(A)** Tumor volume growth curves **(B)** optothermal response **(C)** tumor photo, and **(D)** tumor weight of LLC tumor-bearing mice after systemic administration of saline, blank NPs, free Cino (1 mg/kg), Cino-loaded PDA nanomedicine (1 mg/kg of Cino), and Cino-loaded PDA nanomedicine (1 mg/kg of Cino) treated with 808 NIR laser (2 W cm^−2^, 5 min). Data are presented as the mean ± SD (standard deviation, *n* = 6), *p < 0.05, **p < 0.01.

The anti-tumor efficacy of the blank PDA nanoplatform, free Cino, PDA nanomedicine, and PDA nanomedicine with PTT was studied in LLC tumor-bearing mice. During the monitoring period, neither mouse death nor a significant drop in body weight was observed in any group (Supplementary Figure S8, Supplementary Material), indicating that the treatments did not produce serious toxicity and side effects in the tumor-bearing mice. When the LLC subcutaneous xenograft reached 50 mm^3^ in size, mice were randomly divided into five groups of six mice per group. The mice were then administered saline only, blank PDA NPs (∼5 mg/kg), free Cino (1 mg/kg), PDA nanomedicine (1 mg/kg), and PDA nanomedicine (1 mg/kg) with NIR laser (2 W cm^−2^, 5 min, after treatment) by intraperitoneal injection every alternate day. Before each treatment, body weight and tumor volume were measured. As shown in Figure 6A, the blank PDA NP group showed a similar tendency to that of the control group, indicating the biocompatibility of the PDA nanocarrier. Free Cino effectively inhibited tumor growth. In contrast, PDA nanomedicine had a better therapeutic efficacy than free Cino. Importantly, the PDA nanomedicine combined with laser irradiation at 808 nm almost suppressed the growth of subcutaneous tumors. As shown in Figures 6C,D, photos and the weight of tumors support the results of tumor volume (Figure 6A), clearly showing that free Cino, PDA nanomedicine, and PDA nanomedicine with NIR laser could effectively inhibit tumor growth, with inhibition rates of 29, 48, and 67% on the 11th day, respectively. The temperature increase in the tumor region during NIR laser irradiation is shown in Figure 6B. For the groups treated with saline and free Cino, the temperature increased to 35.2 and 34.9°C, respectively, after irradiation for 5 min (2 W cm^−2^). Conversely, the temperature increased to 41.7, 42.3, and 42.7°C for the groups treated with blank PDA NPs, PDA nanomedicine, and PDA nanomedicine with NIR laser, respectively. These results revealed that the PDA nanomedicine with NIR laser inhibits tumor development most effectively, which is due to the targeted delivery and low pH level stimuli of the PDA nanomedicine in the tumor microenvironment. In addition, the thermal ablation of laser irradiation also increases the therapeutic effect of the PDA nanomedicine.

**FIGURE 7:**
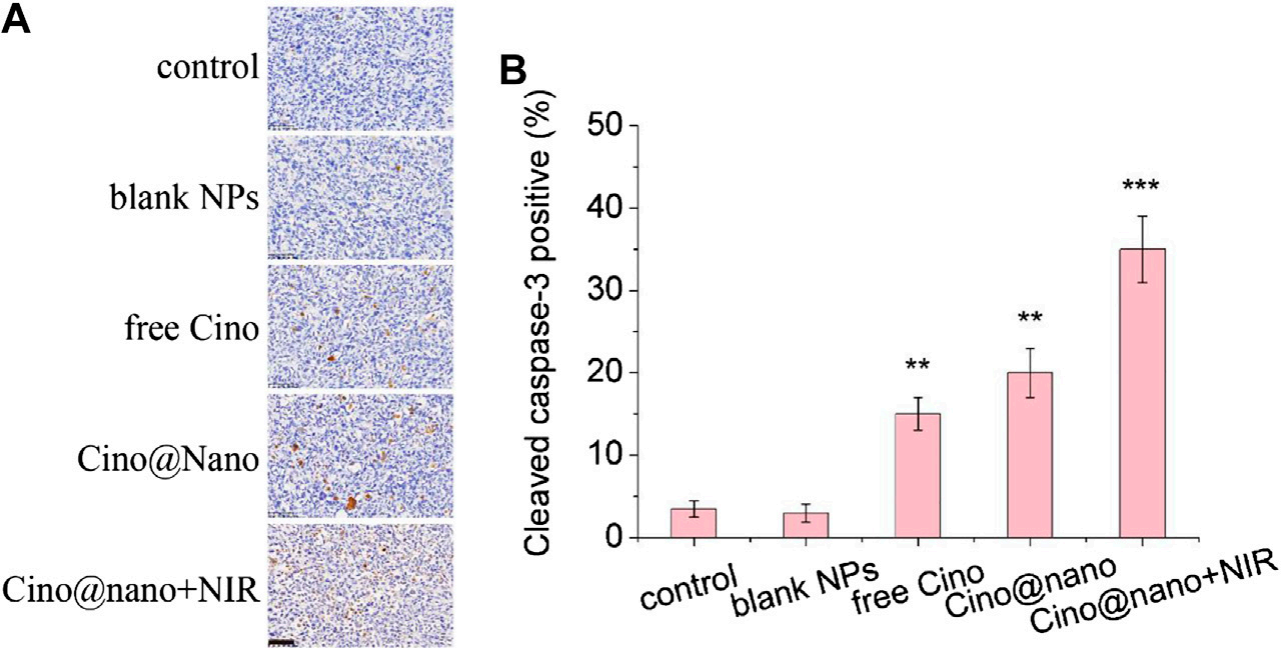
Immunohistochemical staining. **(A)** Cleaved caspase-3 immunohistochemical staining of LLC tumor-bearing mice after systemic administration of saline, blank NPs, free Cino, Cino loaded nanomedicine, and Cino loaded nanomedicine with NIR laser (scale bar = 50 μm). **(B)** Statistical analysis of immunohistochemical staining. Data are presented as the mean ± SD (standard deviation, *n* = 15), **p < 0.01, ***p < 0.001.

To further determine the anticancer efficacy of the PDA nanomedicine with NIR laser, immunohistochemical analysis was also performed. As shown in Figures 7A,B, compared with the control group, the blank PDA NPs showed similar results for the apoptosis factors (cleaved caspase-3). However, free Cino treatment showed significantly increased positive staining for cleaved caspase-3. In addition, Cino-loaded nanomedicine showed more positive staining of cleaved caspase-3 than free Cino. Notably, compared with the tumors treated with Cino@nano, the groups treated with Cino@nano plus NIR irradiation showed typical features of thermal damage in tumor tissues, and possessed the largest number of apoptotic cells, demonstrating the most significant anti-tumor activity of Cino@nano under NIR laser irradiation.

Furthermore, hepatorenal toxicity of the PDA nanomedicine was assessed in terms of ALT, AST, and CRE in mouse serum. As shown in Supplementary Figure S9 (Supplementary Material), the free Cino had the highest ALT, AST, and CRE levels, while the PDA nanomedicine and the PDA nanomedicine with NIR laser had a lesser influence on the liver function of the mice compared with free Cino; the blank NPs had no significant influence. These results indicated that PDA is biodegradable, and that the PDA nanomedicine has properties conducive for targeted delivery and smart response in the tumor microenvironment. The low hepatorenal toxicity of the PDA nanomedicine with PTT is also demonstrated. Altogether, the PDA nanomedicine with NIR laser possesses a significant therapeutic effect and low hepatorenal toxicity.

## Conclusion

A targeting molecule-modified multifunctional drug delivery platform was designed to improve the therapeutic effect of an anticancer agent. PDA nanomedicine can be delivered to tumor cells through FA and FR-mediated cellular endocytosis. In addition, pH-responsive and NIR irradiation-triggered drug release was observed. Both *in vitro* and *in vivo* studies showed that the PDA nanomedicine exerted excellent multimodal (anticancer agent and photothermal) therapeutic effects in inhibiting tumor cell proliferation. This nanomedicine delivery platform is biocompatible and biodegradable due to natural melanin. Importantly, other chemotherapeutic agents and a combination of multiple anticancer drugs as well as genetic agents can be selectively delivered by this smart, responsive, multifunctional nanocarrier. Furthermore, because of the easy introduction of other functional modules onto the surface of the PDA nanomedicine, this work opens up a new avenue to tailor precise PTT nanosystems with high drug accumulation in tumor tissue for a specific patient or disease. Altogether, the present study illustrates the great potential of NIR-responsive and targeted delivery PDA nanomedicine for fast *in situ* drug release to achieve augmented cancer therapy.

## Data Availability

The original contributions presented in the study are included in the article/Supplementary Material, further inquiries can be directed to the corresponding author.
