# Supplementary material for: Cinobufagin-Loaded and Folic Acid-Modified Polydopamine Nanomedicine Combined With Photothermal Therapy for the Treatment of Lung Cancer
Source: Front Chem. 2021 Mar 29;9:637754. doi: 10.3389/fchem.2021.637754 (PMC8039290; doi:10.3389/fchem.2021.637754)
Supplement: Supplementary file 1 [file table1.docx]

**Supporting Information**

**Cinobufagin-loaded and folic acid modified polydopamine nanomedicine with photothermal therapy for the treatment of lung cancer**

Jianwen Li^1#^, Zhanxia Zhang^2#*^, Haibin Deng^1^, Zhan Zheng^1*^

1. Department of Oncology, Longhua Hospital, Shanghai University of Traditional Chinese Medicine, 725 Wanping South Road, Shanghai, 200032, China

2. Cancer Institute, Longhua Hospital, Shanghai University of Traditional Chinese Medicine, 725 Wanping South Road, Shanghai, 200032, China

^#^ These authors contributed equally to this work.

* **Correspondence:**

Zhanxia Zhang, zhanxiazhang@shutcm.edu.cn

Zhan Zheng, zhengzhan@shutcm.edu.cn

**Keywords:** anticancer nanomedicine; photothermal therapy; targeted delivery; stimuli response; biodegradation





**Figure S1.** Zeta potentials of the PDA NPs and Cino-loaded PDA nanomedicine. Error bars represent the mean ± SD (standard deviation, n = 3).





**Figure S2**. FTIR spectra of PDA NPs, FA and FA modified PDA NPs.


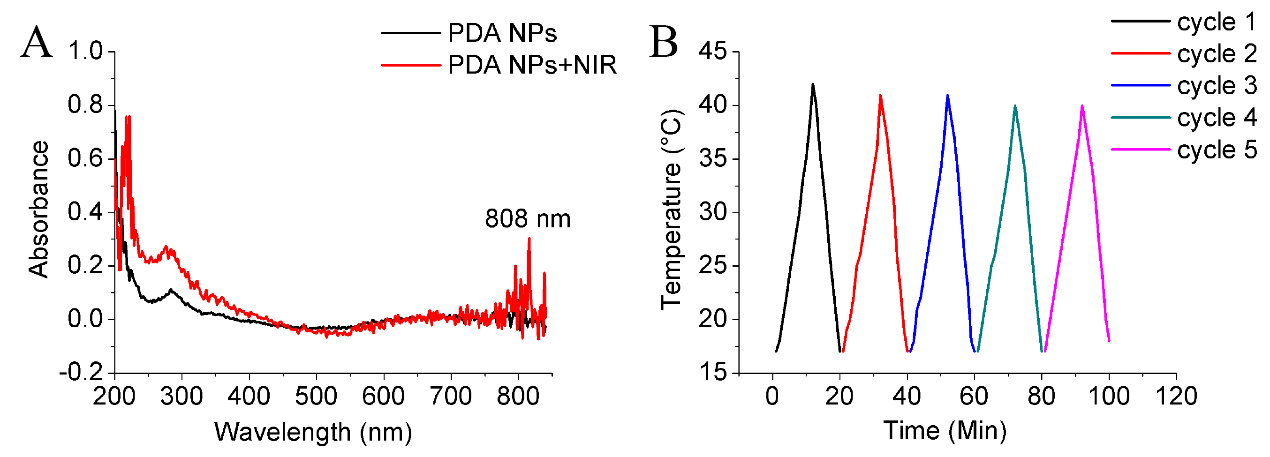


**Figure S3**. (A) The absorption spectrum of the PDA NPs (0.1 mg/ml) with/without NIR irradiation. (B) The photostability of the PDA NPs.


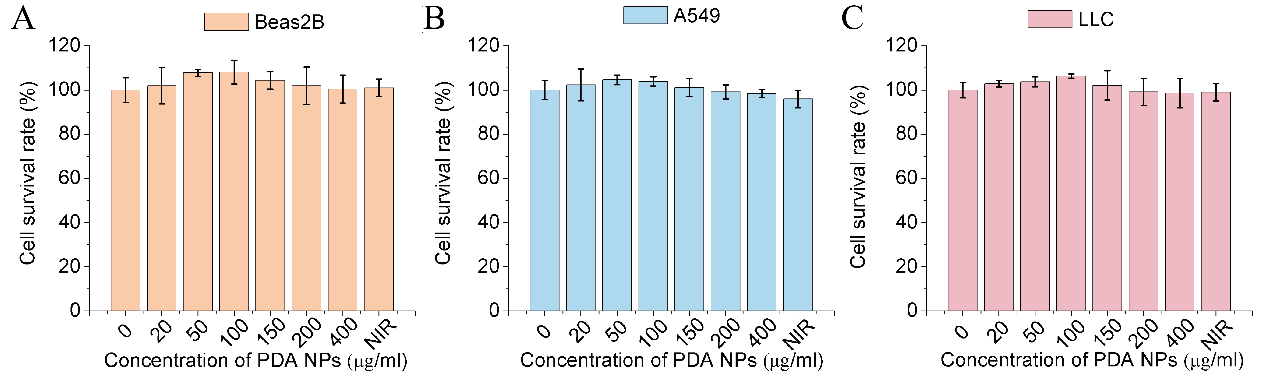


**Figure S4**. Biocompatibility of blank PDA NPs and 808 laser. Cell survival rate of (A) normal bronchial epithelial Beas2B, lung cancer (B) A549 and (C) LLC cells after treating with different amounts of blank PDA NPs and 808 laser (2 W∙cm^-2^, 5 min). Error bars represent mean ± SD (standard deviation, n = 4).


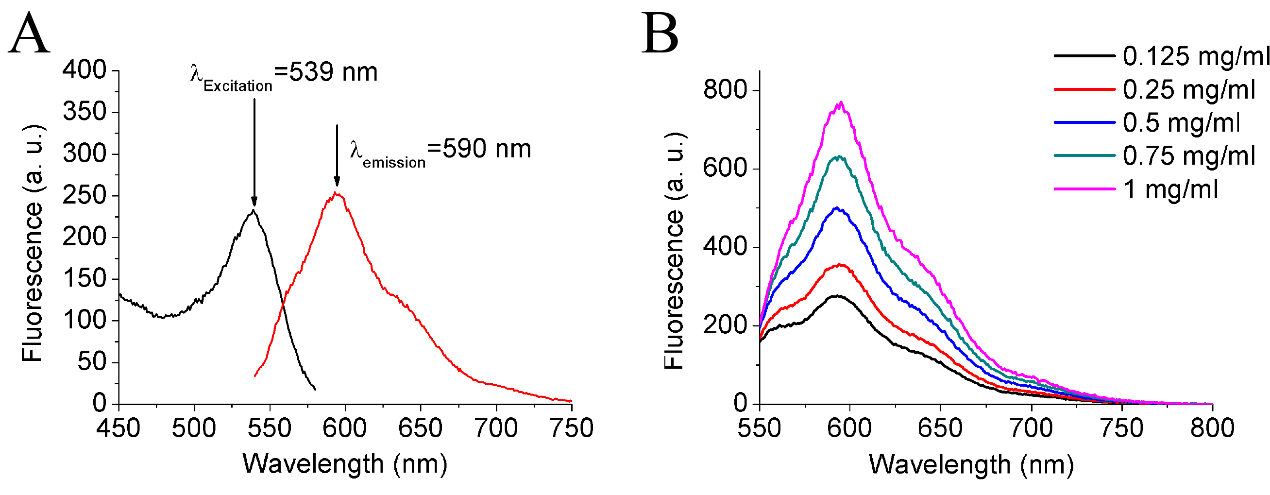


**Figure S5**. Fluorescence images of DOX. (A) Excitation and emission spectra of DOX. (B) The fluorescence curves of different concentration of DOX.





**Figure S6**. *In vitro* anti-tumor efficacy of the blank PDA NPs with NIR treatment in lung cancer (A) A549 cells and (B) LLC cells. Data have been presented as the mean ± SD (standard deviation, n = 4).


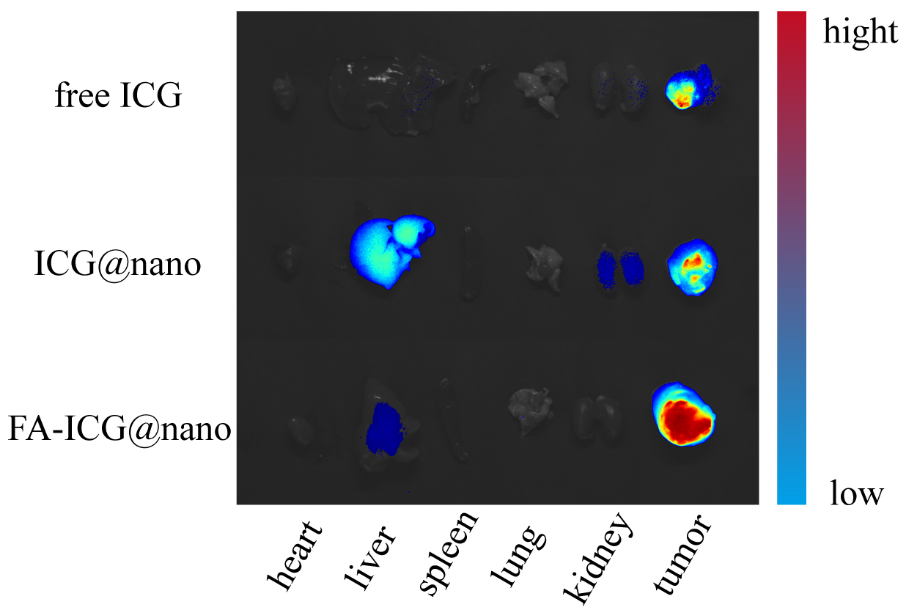


**Figure S7**. Near-infrared (NIR) fluorescence *in vivo* image of indocyanine green (ICG) was observed after 24 h of the intraperitoneal injection of free indocyanine green (ICG), ICG-loaded PDA nanomedicine, and ICG-loaded FA-modified PDA nanomedicine.





**Figure S8**. Body weights changes of LLC tumor bearing mice after systemic administration of saline, blank NPs, free Cino (1 mg/kg), Cino-loaded PDA nanomedicine (1 mg/kg of Cino), and Cino-loaded PDA nanomedicine (1 mg/kg of Cino) with 808 NIR laser (2 W∙cm^-2^, 5 min), error bars represent mean ± SD (standard deviation, n = 6).


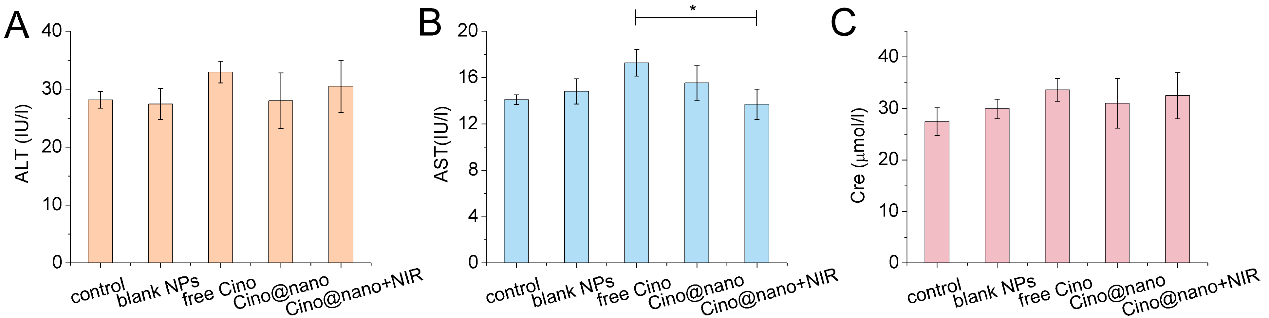


**Figure S9**. Changes in hepatorenal function indices. (A) AST (aspartate transaminase), (B) ALT (alanine transaminase) and CRE (creatinine) values when treated with saline, blank NPs, free Cino (1 mg/kg), Cino-loaded PDA nanomedicine (1 mg/kg of Cino), and Cino-loaded PDA nanomedicine (1 mg/kg of Cino) with 808 NIR laser (2 W∙cm^-2^, 5 min), error bars represent mean ± SD (standard deviation, n = 6), *p< 0.05.
